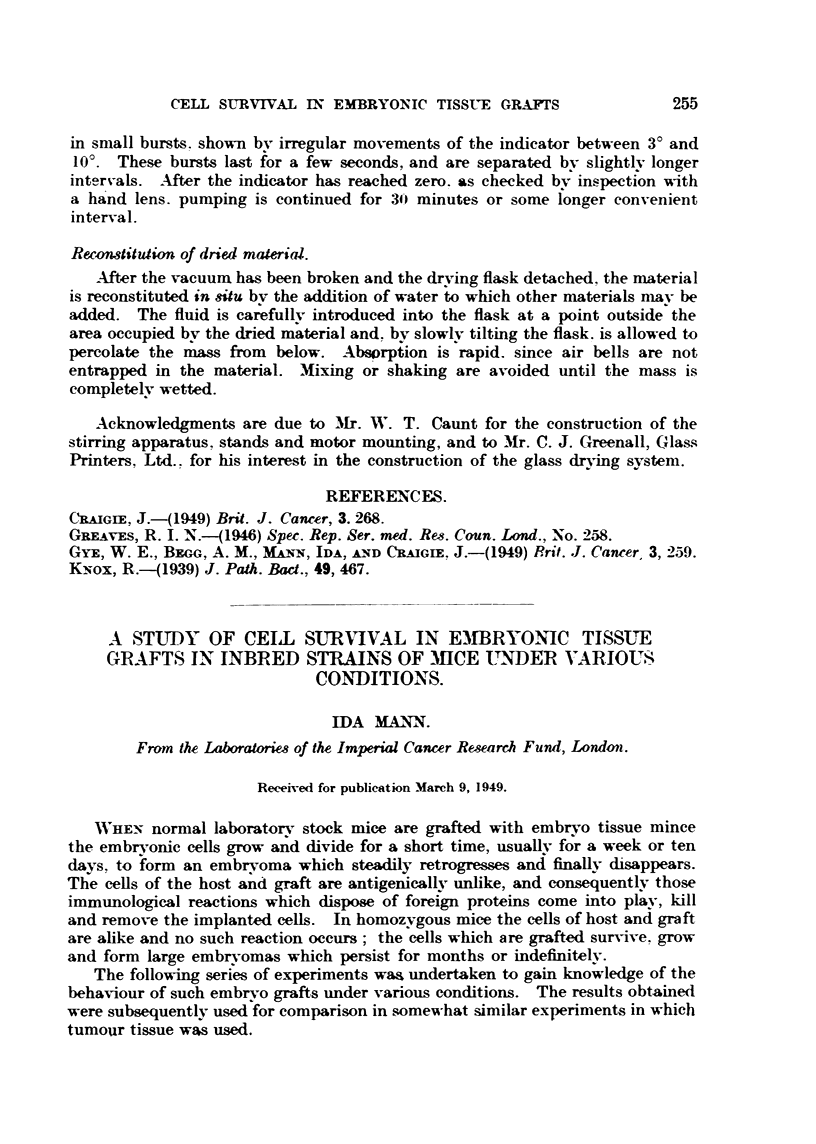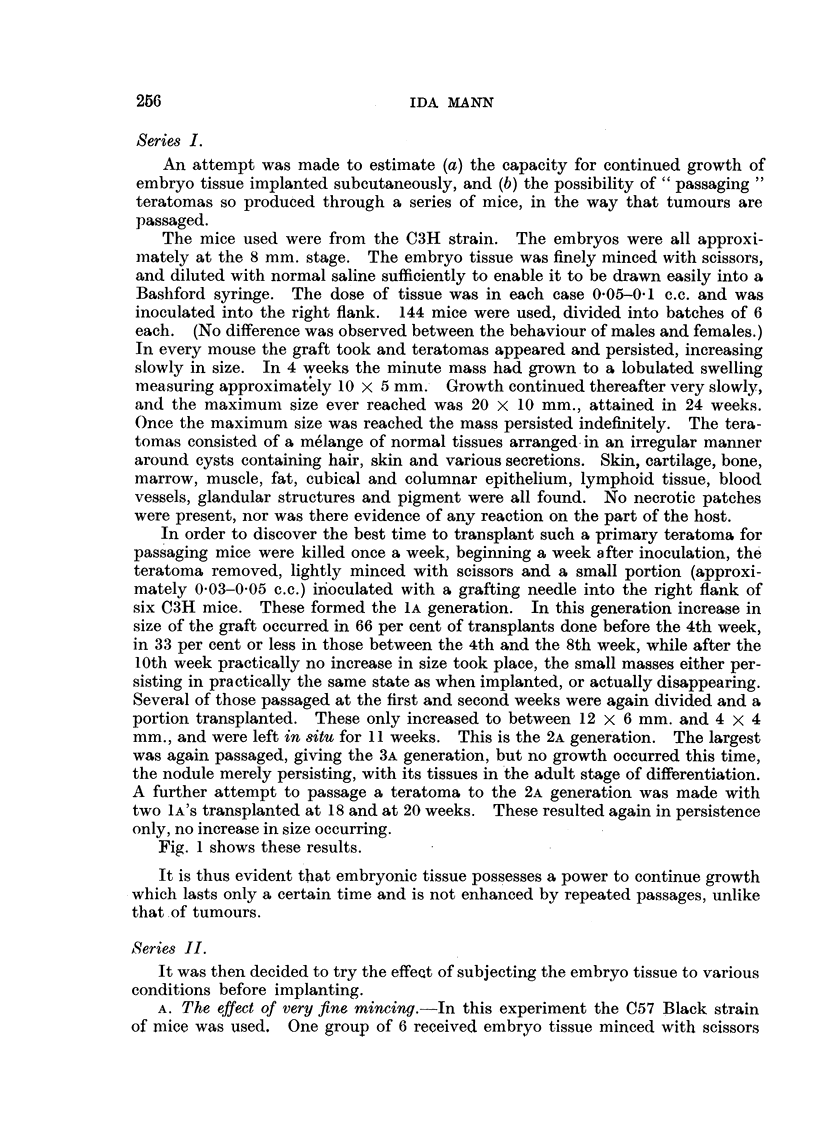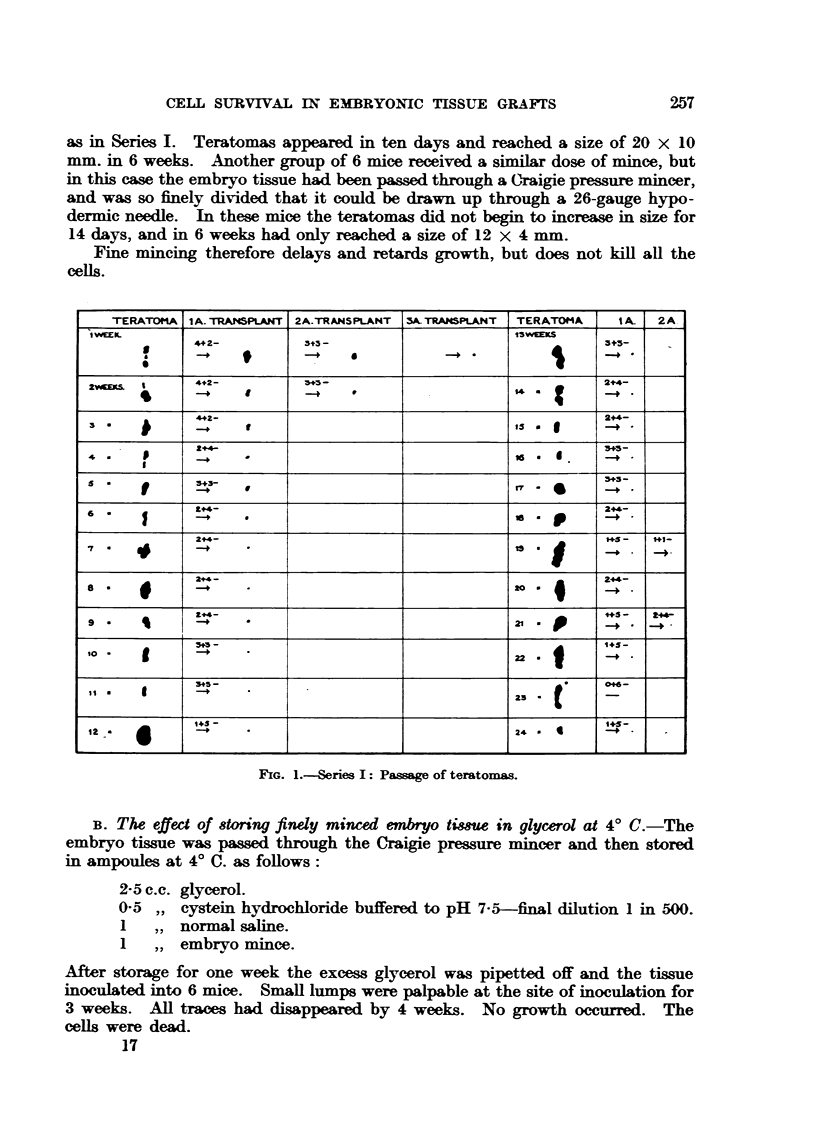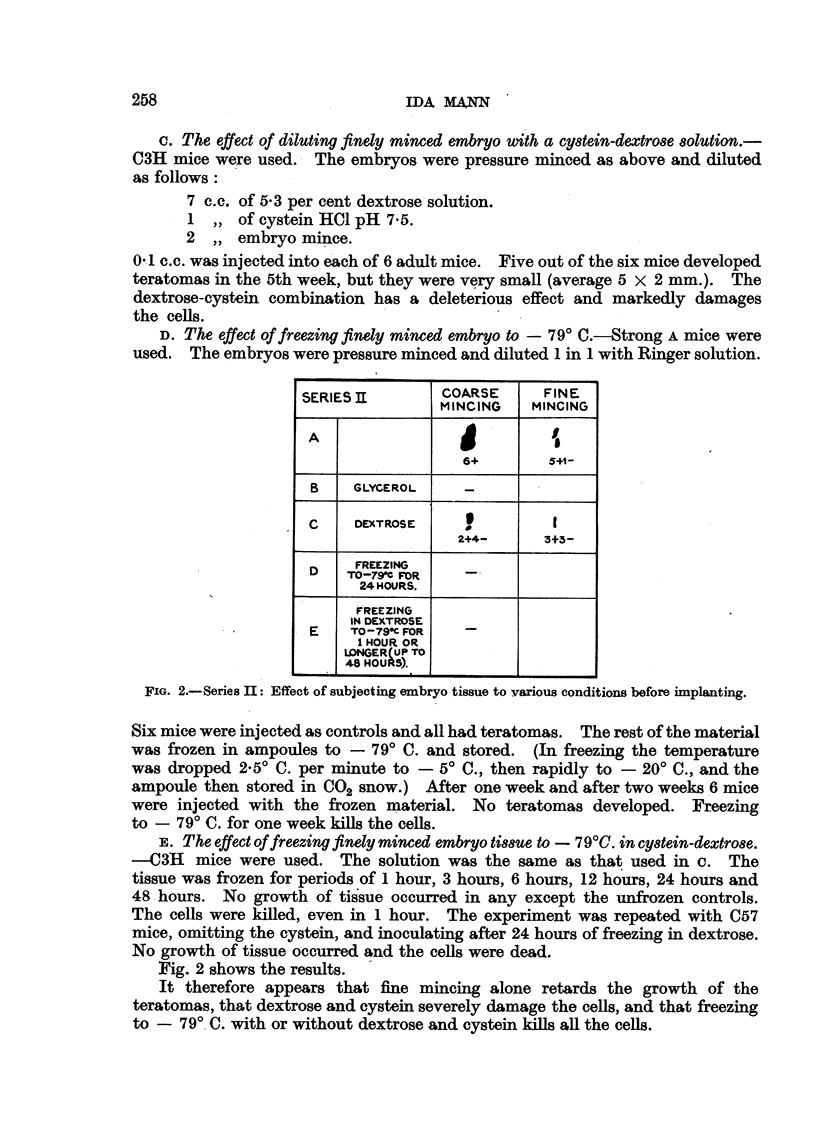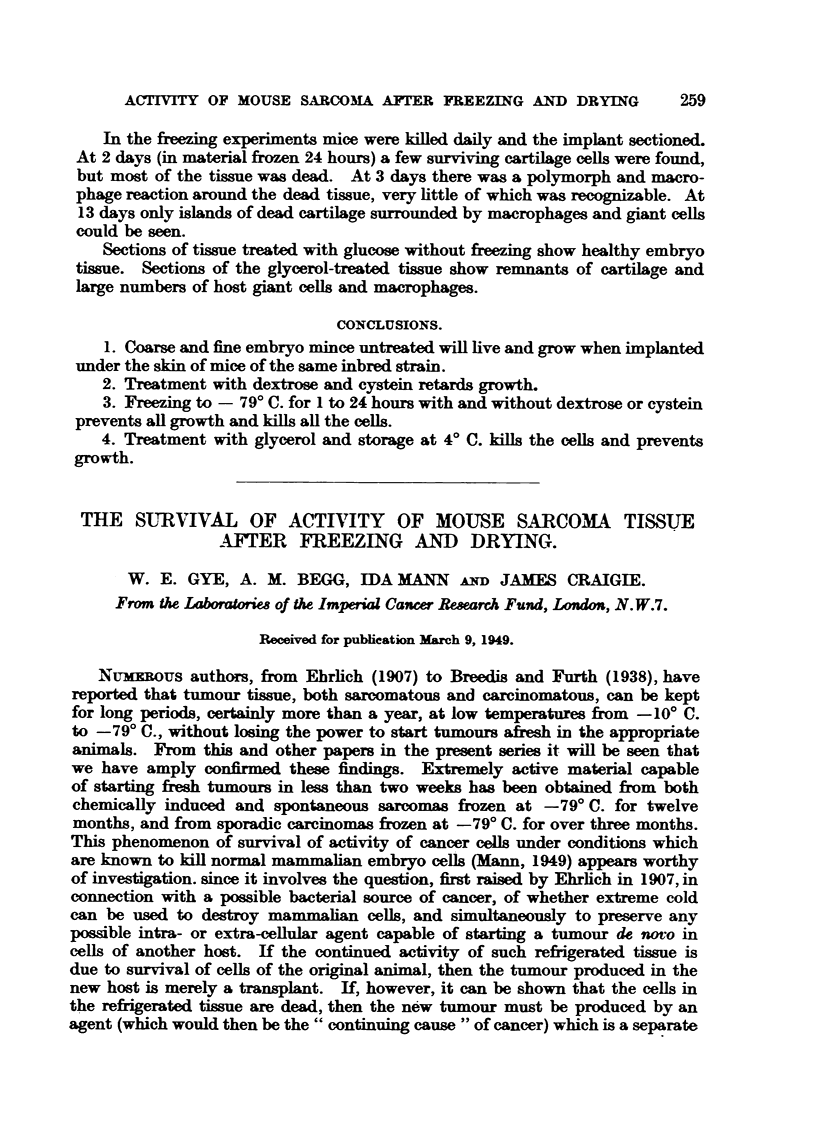# A Study of Cell Survival in Embryonic Tissue Grafts in Inbred Strains of Mice under Various Conditions

**DOI:** 10.1038/bjc.1949.28

**Published:** 1949-06

**Authors:** Ida Mann


### A STUDY OF CELL SURVIVAL IN EMBRYONIC TISSUE GRAFTS IN INBRED STRAINS OF MICE UNDER VARIOUS CONDITIONS.

IDA MANN.

*From the Laboratories of the Imperial Cancer Research Fund, London.*

Received for publication March 9, 1949.

WHEN normal laboratory stock mice are grafted with embryo tissue mince the embryonic cells grow and divide for a short time, usually for a week or ten days, to form an embryoma which steadily retrogresses and finally disappears. The cells of the host and graft are antigenically unlike, and consequently those immunological reactions which dispose of foreign proteins come into play, kill and remove the implanted cells. In homozygous mice the cells of host and graft are alike and no such reaction occurs ; the cells which are grafted survive, grow and form large embryomas which persist for months or indefinitely.

The following series of experiments was undertaken to gain knowledge of the behaviour of such embryo grafts under various conditions. The results obtained were subsequently used for comparison in somewhat similar experiments in which tumour tissue was used.

*Series I.*

An attempt was made to estimate (*a*) the capacity for continued growth of embryo tissue implanted subcutaneously, and (*b*) the possibility of " passaging " teratomas so produced through a series of mice, in the way that tumours are passaged.

The mice used were from the C3H strain. The embryos were all approximately at the 8 mm. stage. The embryo tissue was finely minced with scissors, and diluted with normal saline sufficiently to enable it to be drawn easily into a Bashford syringe. The dose of tissue was in each case 0·05–0·1 c.c. and was inoculated into the right flank. 144 mice were used, divided into batches of 6 each. (No difference was observed between the behaviour of males and females.) In every mouse the graft took and teratomas appeared and persisted, increasing slowly in size. In 4 weeks the minute mass had grown to a lobulated swelling measuring approximately 10 × 5 mm. Growth continued thereafter very slowly, and the maximum size ever reached was 20 × 10 mm., attained in 24 weeks. Once the maximum size was reached the mass persisted indefinitely. The teratomas consisted of a mélange of normal tissues arranged in an irregular manner around cysts containing hair, skin and various secretions. Skin, cartilage, bone, marrow, muscle, fat, cubical and columnar epithelium, lymphoid tissue, blood vessels, glandular structures and pigment were all found. No necrotic patches were present, nor was there evidence of any reaction on the part of the host.

In order to discover the best time to transplant such a primary teratoma for passaging mice were killed once a week, beginning a week after inoculation, the teratoma removed, lightly minced with scissors and a small portion (approximately 0·03–0·05 c.c.) inoculated with a grafting needle into the right flank of six C3H mice. These formed the 1A generation. In this generation increase in size of the graft occurred in 66 per cent of transplants done before the 4th week, in 33 per cent or less in those between the 4th and the 8th week, while after the 10th week practically no increase in size took place, the small masses either persisting in practically the same state as when implanted, or actually disappearing. Several of those passaged at the first and second weeks were again divided and a portion transplanted. These only increased to between 12 × 6 mm. and 4 × 4 mm., and were left *in situ* for 11 weeks. This is the 2A generation. The largest was again passaged, giving the 3A generation, but no growth occurred this time, the nodule merely persisting, with its tissues in the adult stage of differentiation. A further attempt to passage a teratoma to the 2A generation was made with two 1A's transplanted at 18 and at 20 weeks. These resulted again in persistence only, no increase in size occurring.

Fig. 1 shows these results.

It is thus evident that embryonic tissue possesses a power to continue growth which lasts only a certain time and is not enhanced by repeated passages, unlike that of tumours.

*Series II.*

It was then decided to try the effect of subjecting the embryo tissue to various conditions before implanting.

A. *The effect of very fine mincing.*—In this experiment the C57 Black strain of mice was used. One group of 6 received embryo tissue minced with scissors as in Series I. Teratomas appeared in ten days and reached a size of 20 × 10 mm. in 6 weeks. Another group of 6 mice received a similar dose of mince, but in this case the embryo tissue had been passed through a Craigie pressure mincer, and was so finely divided that it could be drawn up through a 26-gauge hypodermic needle. In these mice the teratomas did not begin to increase in size for 14 days, and in 6 weeks had only reached a size of 12 × 4 mm.

Fine mincing therefore delays and retards growth, but does not kill all the cells.

FIG. 1.—Series I: Passage of teratomas.

B. *The effect of storing finely minced embryo tissue in glycerol at 4° C.*—The embryo tissue was passed through the Craigie pressure mincer and then stored in ampoules at 4° C. as follows :

2·5 c.c. glycerol.
0·5 ,, cystein hydrochloride buffered to pH 7·5—final dilution 1 in 500.
1 ,, normal saline.
1 ,, embryo mince.

After storage for one week the excess glycerol was pipetted off and the tissue inoculated into 6 mice. Small lumps were palpable at the site of inoculation for 3 weeks. All traces had disappeared by 4 weeks. No growth occurred. The cells were dead.

C. *The effect of diluting finely minced embryo with a cystein-dextrose solution.*—C3H mice were used. The embryos were pressure minced as above and diluted as follows:

7 c.c. of 5·3 per cent dextrose solution.

1 „ of cystein HCl pH 7·5.

2 „ embryo mince.

0·1 c.c. was injected into each of 6 adult mice. Five out of the six mice developed teratomas in the 5th week, but they were very small (average 5 × 2 mm.). The dextrose-cystein combination has a deleterious effect and markedly damages the cells.

D. *The effect of freezing finely minced embryo to — 79° C.*—Strong A mice were used. The embryos were pressure minced and diluted 1 in 1 with Ringer solution.

| SERIES II | | COARSE MINCING | FINE MINCING |
|---|---|---|---|
| A | | 6+ | 5+1− |
| B | GLYCEROL | — | |
| C | DEXTROSE | 2+4− | 3+3− |
| D | FREEZING TO−79°C FOR 24 HOURS. | — | |
| E | FREEZING IN DEXTROSE TO−79°C FOR 1 HOUR OR LONGER (UP TO 48 HOURS). | — | |

FIG. 2.—Series II: Effect of subjecting embryo tissue to various conditions before implanting.

Six mice were injected as controls and all had teratomas. The rest of the material was frozen in ampoules to — 79° C. and stored. (In freezing the temperature was dropped 2·5° C. per minute to — 5° C., then rapidly to — 20° C., and the ampoule then stored in $CO_2$ snow.) After one week and after two weeks 6 mice were injected with the frozen material. No teratomas developed. Freezing to — 79° C. for one week kills the cells.

E. *The effect of freezing finely minced embryo tissue to — 79°C. in cystein-dextrose.*—C3H mice were used. The solution was the same as that used in C. The tissue was frozen for periods of 1 hour, 3 hours, 6 hours, 12 hours, 24 hours and 48 hours. No growth of tissue occurred in any except the unfrozen controls. The cells were killed, even in 1 hour. The experiment was repeated with C57 mice, omitting the cystein, and inoculating after 24 hours of freezing in dextrose. No growth of tissue occurred and the cells were dead.

Fig. 2 shows the results.

It therefore appears that fine mincing alone retards the growth of the teratomas, that dextrose and cystein severely damage the cells, and that freezing to — 79° C. with or without dextrose and cystein kills all the cells.

In the freezing experiments mice were killed daily and the implant sectioned. At 2 days (in material frozen 24 hours) a few surviving cartilage cells were found, but most of the tissue was dead. At 3 days there was a polymorph and macrophage reaction around the dead tissue, very little of which was recognizable. At 13 days only islands of dead cartilage surrounded by macrophages and giant cells could be seen.

Sections of tissue treated with glucose without freezing show healthy embryo tissue. Sections of the glycerol-treated tissue show remnants of cartilage and large numbers of host giant cells and macrophages.

### CONCLUSIONS.

1. Coarse and fine embryo mince untreated will live and grow when implanted under the skin of mice of the same inbred strain.

2. Treatment with dextrose and cystein retards growth.

3. Freezing to — 79° C. for 1 to 24 hours with and without dextrose or cystein prevents all growth and kills all the cells.

4. Treatment with glycerol and storage at 4° C. kills the cells and prevents growth.

---